# Increased Cardiovascular and Renal Risk Is Associated with Low Nephron Endowment in Aged Females: An Ovine Model of Fetal Unilateral Nephrectomy

**DOI:** 10.1371/journal.pone.0042400

**Published:** 2012-08-03

**Authors:** Reetu R. Singh, Andrew J. Jefferies, Yugeesh R. Lankadeva, Paul Lombardo, Michal Schneider-Kolsky, Lucinda Hilliard, Kate M. Denton, Karen M. Moritz

**Affiliations:** 1 Department of Anatomy and Developmental Biology, Monash University, Victoria, Australia; 2 Department of Physiology, Monash University, Victoria, Australia; 3 Department of Medical Imaging and Radiation Sciences, Monash University, Victoria, Australia; 4 School of Biomedical Sciences, University of Queensland, St Lucia, Australia; The University of Manchester, United Kingdom

## Abstract

Previously we have shown that ovariectomised (OVX) female sheep have reduced renal function and elevated blood pressure from 6 months of age following fetal uninephrectomy (uni-x) at 100 days of gestation (term  = 150 days). In the current study we examined if in intact female sheep the onset of decline in renal function and elevation in blood pressure was prevented. Studies were performed at 1 year, 2 and 5 years of age. Following fetal uni-x at 100 days, intact female sheep had ∼30% reduction in glomerular filtration rate (GFR) at 1 year, which did not exacerbate with age (P_treatment_ = 0.0001, P_age_ = 0.7). In contrast renal blood flow was similar between the treatment groups at 1 year of age but had declined in the uni-x animals at 5 years of age (P_treatment × age_ = 0.046). Interestingly, intact uni-x sheep did not develop elevations in arterial pressure until 2 years of age. Furthermore, uni-x animals had a similar capacity to respond to a cardiac challenge at 1 year and 2 years of age, however, cardiac functional reserve was significantly reduced compared to sham group at 5 years of age. Uni-x animals exhibited an increase in left ventricular dimensions at 5 years of age compared to the sham animals and compared to 2 years of age (P_treatment_<0.001, P_treatment × age_<0.001). In conclusion, the onset of renal dysfunction preceded the onset of hypertension in intact female uni-x sheep. Furthermore, this study showed that the intact females are protected from the impact of a reduced nephron endowment on cardiovascular health early in life as opposed to our findings in young male sheep and OVX uni-x female sheep. However, with ageing this protection is lost as evidenced by presence of left ventricular hypertrophy and impaired cardiac function in 5 year old uni-x female sheep.

## Introduction

Numerous studies have shown sex differences in the incidence and progression of cardiovascular and kidney disease [1], [2] with a higher risk observed in males compared to age-matched premenopausal women [3]. A congenital nephron deficit has been proposed to be a risk factor for hypertension in adulthood due to renal insufficiency [4]. A number of animal studies have shown that alterations in renal development resulting in reduced nephron number in the offspring, as occur following fetal growth restriction due to maternal dietary protein restriction [5], [6], uteroplacental insufficiency [7], exposure of the developing fetus to elevation in maternal stress hormones, glucocorticoids [8], [9], [10], [11] or prenatal alcohol exposure [12], result in blood pressure elevation in these offspring in adulthood.

More importantly, there is now significant evidence to suggest that sex-differences also exist in the developmental programming of adult disease, with females being relatively protected [13], [14], [15]. For example, following a modest dietary protein restriction in rats (8.5% instead of 19% protein) during the entire pregnancy, only male offspring have a reduction in nephron number and develop hypertension [16]. In the model of uteroplacental insufficiency, only growth restricted male rat offspring become hypertensive after puberty [17], whereas, female offspring which are ovariectomised (OVX) in that model develop hypertension post-puberty which can be reversed by estrogen [18]. The mechanisms responsible for these sex related differences are not clearly understood. However, both female (estrogen and progesterone) as well as male (testosterone) hormones likely play a role [19]. While estrogen is acknowledged to account for sex-specific differences in cardiovascular function, hormone/estrogen replacement therapy (HRT), expected to be cardio-protective, has proven controversial [20]. The Nurse’s Health Study provided the first report of reduction in cardiovascular events in women on HRT [21]. However, the Heart Estrogen/Progestin Replacement Study, the first prospective, controlled study to evaluate HRT did not demonstrate a reduction in cardiovascular risk and moreover, indicated an increased risk in the first year of therapy [20]. Furthermore, estrogen has not always been shown to be beneficial in the kidney either. Obese female Zucker rats have a greater progression of chronic kidney disease than male rats [22] and estradiol has been shown to increase stroke and renal injury in the stroke-prone hypertensive rats [23].

Our group has previously reported that a reduction in nephron number at birth induced by fetal unilateral nephrectomy (uni-x) at 100 days of gestation (term  = 150 days) results in reduced kidney function and elevation in arterial pressure in both male [24], [25], [26] and female [27] sheep from 6 months of age. However, a caveat to our previous studies in female sheep was that OVX was performed early in life to minimise the effect of the estrous cycle on blood pressure levels [27], [28]. Therefore, in the current study we firstly examined if intact uni-x female sheep were protected from the adverse cardiovascular and renal effects of a reduced nephron endowment. After the onset of hypertension was determined in the present study, we examined if the elevation in arterial pressure resulted in the common sequelae of left ventricular hypertrophy. Furthermore, since cardiac functional capacity is significantly attenuated in the uni-x male sheep from as early as 6 months of age [24], we wanted to establish if a similar cardiac defect existed in the uni-x female sheep. We hypothesised that the presence of estrogen would delay the onset of hypertension in the uni-x sheep. In addition, it was hypothesised that renal function (glomerular filtration rate and renal blood flow) at 1 year of age would be similar to sham animals despite the reduction in nephron number due to an increase in filtration fraction, indicating glomerular hyperfiltration in the uni-x animals.

## Materials and Methods

### Cohort Set-up

All experiments were approved by an Animal Ethics Committee of Monash University and were performed in accordance with the guidelines of the National Health and Medical Research Council of Australia. Merino ewes carrying single female fetus’ of known gestational age underwent surgery at 100 days post conception. Anaesthesia was induced in ewes and fetus’ with sodium pentothal (1 g I.V.) and maintained with halothane (1.5–2% in O_2_). The fetal left kidney was cleared from surrounding fat and the left renal artery, left renal vein and ureter were ligated (uni-x group  = 5) and the kidney excised. In 5 fetuses, the kidney was cleared from the surrounding fat but was not excised (sham-operated group  = 5). Post surgery, ewes were housed in large pens in the animal house for 2 weeks, before being sent to a farm for the remainder of pregnancy. After birth, lambs remained with their mothers on pasture until 18 weeks of age when they weaned. At 5 months of age the lambs underwent surgery and the right carotid artery was surgically exteriorised into a skin fold to form a carotid arterial loop [29]. At 1 year, 2 and 5 years of age, animals were brought into the laboratory, placed in individual metabolic cages and allowed a week to acclimatise to laboratory conditions. All animals were maintained on a diet of hay and chaff for the duration of their stay in the laboratory. Following the acclimatisation period, animals were instrumented with a carotid arterial catheter (Tygon cannula) for measurement of arterial pressure and a venous catheter (right jugular vein) for infusion purposes.

### Plasma Ion and Hormone Levels at 1, 2 and 5 Years of Age

Basal arterial blood samples were collected at 1 year, 2 and 5 years of age for determination of plasma sodium via Beckman Synchron CX-5 clinical system, (Beckman Instruments Inc), plasma renin activity (PRA) by radioimmunoassay (Prosearch International Australia) and plasma 17β-estradiol via a commercially available kit (Ultra-sensitive estradiol RIA, Beckman Coulter).

### Basal 72 Hour Mean Arterial Pressure and Heart Rate at 1, 2 and 5 Years of Age

For measurement of conscious mean arterial pressure (MAP) and heart rate (HR), a tygon cannula was inserted into the carotid arterial loop. Baseline MAP and HR measurements were acquired every 10 seconds and averaged every 10 minutes, over a 72 hour period and cumulative averages of these are reported as basal MAP and HR.

### Blood Volume Measurement Using Fluorescein Isothiocyanate (FITC)-dextran

Following 72 hour blood pressure recordings, blood and plasma volume measurements were obtained by determining dextran space using 250 kDa FITC-dextran (Sigma, St Louis, USA) as previously described [30].

### Renal Function at 1 Year and 5 Years of Age

Forty-eight hours after the blood volume measurements were completed, a Foley catheter, (Size 12, French, Bardia Malaysia) was inserted into the bladder of all animals for continuous collection of urine. GFR was determined over a 3 hour collection period via the clearance of ^Cr^51-ethylenediaminetetraacetic acid (EDTA, Amersham International) and effective renal plasma flow (ERPF), and hence RBF, were determined via clearance of para-aminohippurate (PAH) as previously described [26]. PAH concentration was determined using a previously described rapid microplate assay method [31]. Renal vascular resistance (RVR) was determined as [MAP/RBF]. Filtration fraction (%) was determined as [GFR/ERPF]; urinary sodium excretion (U_Na_V) was determined as [urine flow rate (UFR) × urinary sodium concentration]; filtered load of sodium (FL_Na_) was calculated as [plasma sodium concentration × GFR] and fractional sodium excretion (FE_Na_ %) was calculated as [(U_Na_V/filtered load of sodium)×100].

### Cardiac Function at 1, 2 and 5 Years of Age

All measurements of cardiac function (basal 24 hour measurement and response to dobutamine challenge (see sections below) were performed in conscious animals. Forty-eight hours after the completion of the renal function measurements, the animals were cannulated with a Swan-Ganz catheter (Baxter, Australia) as described previously [32] for measurement of cardiac function. Cardiovascular variables (MAP, HR, cardiac output (CO), central venous pressure (CVP), and mean pulmonary artery pressure (PAM), were measured simultaneously and continuously over 24 hours with CO readings obtained from a CO computer (9520A, American Edwards Laboratories). Stroke volume (SV) and total peripheral resistance (TPR) were calculated from the measured variables.

### Cardiac Functional Reserve at 1, 2 and 5 Years of Age

Cardiac functional reserve (maximal response relative to basal levels, CO_max-0_) was determined by evaluating the CO response to β-adrenergic stimulation (dobutamine challenge; Dobutamine hydrochloride, Sigma Aldrich, Australia) where dobutamine was infused in eight incremental doses with the doses ranging from 0.5–7.0 µg/kg/min (10 mins per dose) or until the CO had reached a plateau as described previously in sheep [32]. Briefly, on the day of experimentation, basal MAP, HR, CO, PAM, pulmonary artery wedge pressure (WP), and CVP were measured over one hour. To eliminate any differences in preload pressures between the animals, each animal was infused with a plasma volume expander (Hemaccel; infusion rate 15 ml/min) in 200 ml steps until the WP had reached 10 mmHg. Once the desired WP was reached, MAP_0_, CO_0_, HR_0_, PAM_0_, WP_0_ and CVP_0_ were measured to establish a new baseline prior to the dobutamine challenge. WP was maintained at 10 mmHg for the duration of the experiment.

### Echocardiographic Measurement at 2 and 5 Years of Age

M-mode scanning of the heart was performed in conscious sheep at the short parasternal axis at the level of the papillary muscle HDI 5000 SonoCT ultrasound system with a P5-3 MHz transducer (Philips) [32]. Left ventricular measurements were obtained in both diastole (left ventricular diameter, (LVDd); posterior wall thickness (PWDd); interventricular septal diameter (IVSd) and in systole (left ventricular diameter, (LVDs); posterior wall thickness (PWDs); interventricular septum (IVSDs). Five consecutive cardiac cycles were measured and the averages calculated for each of the aforementioned variables. Left Ventricular mass (LV mass) was determined using the Penn Convention: LV mass  =  1.04((LVD+PWD+IVSd)^3^-(LVD)^3^)-13.6 g and then indexed for animal body weight. Percentage fractional shortening (%FS) and relative wall thickness (RWT) were calculated using the following formulae. %FS = (LVDd-LVDs)/LVDd *100; RWT  =  (PWDd+IVSDd)/LVD [32].

### Cardiac Gene Expression at 5 Years of Age

All animals were humanely euthanized (pentobarbitone, Lethabarb®) one week following the completion of experiments at 5 years of age and hearts were excised. A 0.5 cm thick slice at the level of the apex was frozen for molecular studies and the remaining heart immersion fixed in 10% buffered formalin. The left ventricle and septum from the frozen slices was homogenised and RNA extracted for determining gene expression of collagen I, elastin and transforming growth factor-β1 (TGF-β1) by real-time PCR as previously described [33].

### Cardiac Morphometry-Quantification of Interstitial Collagen at 5 Years of Age

After an appropriate period of fixation, the left ventricle together with septum (LV+S) for the entire heart was sampled into 5 mm thick tranverse sections approximately 10 mm below the plane of the valves. These sections were then sampled using the smooth fractionator approach and selected samples (8–10 pieces) per heart were embedded in paraffin and sectioned at 5 µm. Sections were then stained with picrosirius red after 4 minute pre-treatment with phosphomolybdic acid and interstitial fibrosis assessed by determining percentage of collagen within the interstitium as previously described [34].

### Statistical Analysis

Values are presented as the mean ± SEM, with the level of significance set at less than or equal to 0.05. A two-way repeated analysis of variance (ANOVA) was used to examine the differences between treatment (sham vs uni-x) and age (1 vs 2 vs 5 years) and the interaction between treatment and age. A Bonferroni post-hoc test was performed where appropriate. Statistical analysis was performed using SYSTAT software (SYSTAT 11 for Windows, SPSS Science, UK).

## Results

### Birth Weight and Growth

All animals were born at term (149±1 day). Birth and body weights were similar between both treatment groups at all ages (birth weight (kg): sham; 4.0±0.5, uni-x; 4.0±0.3, 1 year (kg): sham; 40±2; uni-x; 43±1, 2 years (kg): sham; 57±4; uni-x; 59±5, 5 years (kg): sham; 56±2, 54±6). At 5 years of age, there was no difference in total kidney weight between the treatment groups (sham: total (left and right) kidney weight, (g); 121±6, uni-x: right kidney weight (g); 108±6). Total kidney to body weight ratio (g/kg) was also not different between the groups (sham; 2.2±0.1, uni-x; 2.1±0.2).

### Plasma Ions and Hormones

**Figure 1:**
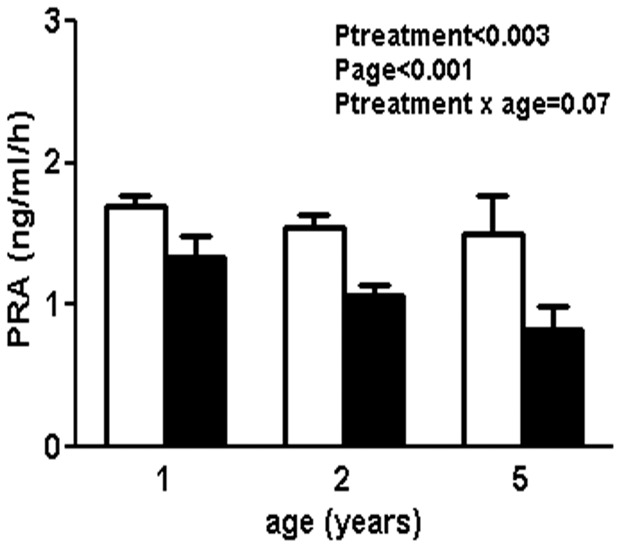
Plasma renin activity (PRA) in sham and uni-x animals (n = 5 in each group) at 1 year, 2 and 5 years of age. Values are from two-way repeated ANOVA and expressed as mean ± SEM. Sham; clear bars, uni-x; dark bars.

Plasma sodium was similar between the treatment groups at all ages studied (Plasma sodium (mmol/l): 1 year; sham: 144±1, uni-x: 143±1, 2 years; sham: 141±2, uni-x: 143±2, 5 years; sham: 143±1, uni-x: 143±1). Plasma 17β-estrodial concentrations were below the detection limit of the assay kit (2.2 pg/ml) for some animals in each treatment group at all ages examined. At 1 year of age, one animal in each treatment group had levels above the sensitivity of the assay (estradiol levels (pg/ml); sham:4.25, uni-x:3.38) whilst no animal in either group had measurable levels at 2 years of age. At 5 years, 3 animals in the sham group had measurable estradiol levels (average  =  3.82±0.3 pg/ml) whilst the other 2 sham animals and the 5 uni-x animals had plasma concentrations below the level of the assay sensitivity. PRA levels were significantly lower in the uni-x compared to sham group at all ages (P_treatment_<0.003). While PRA levels did not differ with ageing in the sham animals, levels tended to decline further with ageing in the uni-x group (P_age_<0.001, P_treatment × age_ = 0.07, Fig. 1).

### Renal Function Measurements at 1 and 5 Years of Age

Renal function measurements are shown in Table 1. GFR (ml/kg/h) was significantly lower in the uni-x compared to the sham group at both ages (P_treatment_<0.0001) and was not altered significantly with ageing in either treatment group (P_age_ = 0.7, P_treatment × age_ = 0.3). RBF was similar between the treatment groups at 1 year of age. However, at 5 years of age, RBF was significantly lower in the uni-x compared to the sham group and compared to RBF in uni-x animals at 1 year of age (P_treatment_ = 0.2, P_age_ = 0.02, P_treatment × age_ = 0.05). RVR was not different between the treatment groups at 1 year of age. However, at 5 years of age, RVR increased significantly in the uni-x group from 1 year age and uni-x animals had a significantly greater RVR compared to sham animals at 5 years of age (P_treatment_ = 0.008, P_age_ = 0.007, P_treatment × age_ = 0.001). Two-Way ANOVA showed a significant effect of treatment and age on filtration fraction (%) and a tendency for an interaction between these two factors (P_treatment_ = 0.03, P_age_ = 0.002, P_treatment × age_ = 0.06). At 1 year of age, filtration fraction was significantly lower in the uni-x animals compared to the sham group (P<0.01, post-hoc test) but the uni-x animals at 5 years of age had similar filtration fraction to their age-matched counterparts. While, filtration fraction increased in both treatment groups with age, the increase in filtration fraction with age was significantly greater in the uni-x animals compared to the sham group (absolute change in filtration fraction: sham; 5.5±0.7, uni-x; 10.6±2.1, P = 0.01 from t-test). UFR was similar between the treatment groups at both ages. However, UFR was significantly but similarly reduced in animals at 5 years compared to 1 year of age (P_treatment_ = 0.8, P_age_ = 0.02, P_treatment × age_ = 0.9). U_Na_V was not different with age and while uni-x animals had reduced U_Na_V, this was not statistically different from the sham group at any age (P_treatment_ = 0.2, P_age_ = 0.2, P_treatment × age_ = 0.4). FL_Na_ was significantly lower in the uni-x animals at both ages compared to the sham group (P_treatment_<0.001, P_age_ = 0.4, P_treatment × age_ = 0.5). Similar to U_Na_V, FE_Na_ % also did not differ with age and while uni-x animals had lower FE_Na_%, this was not statistically different to the sham group at any age (P_treatment_ = 0.1, P_age_ = 0.4, P_treatmentxage_ = 0.5).

**Table 1:** Renal function at 1 year and 5 years of age in intact female sham and uni-x sheep.

	*sham*		*Uni-x*		*P-value*
Age (years)	1 year	5 years	1 year	5 years	
Plasma sodium (mmol)	144±1	143±1	143±1	143±1	P_treatment_ = 0.3 P_age_ = 0.3 P_treatment × age_ = 0.5
UFR (ml/kg/h)	2.0±0.4	0.8±0.2	1.8±0.5	0.9±0.2	P_treatment_ = 0.8 P_age_ = 0.002 P_treatment × age_ = 0.9
U_Na_V (µmol/kg/h)	109±64	192±64	87±49	79±23	P_treatment_ = 0.2 P_age_ = 0.2 P_treatment × age_ = 0.4
Filtered load of sodium(mmol/kg/h)	17.4±1.0	17.3±0.2	11.1±0.3	9.7±0.1	P_treatment_<0.001 P_age_ = 0.4 P_treatment × age_ = 0.5
FE_Na_ %	1.5±0.4	1.3±0.3	0.8±0.4	1.0±0.2	P_treatment_ = 0.1 P_age_ = 0.4 P_treatment × age_ = 0.5
GFR (ml/kg/h)	121±6	125±2	78±2	69±8	P_treatment_<0.001 P_age_ = 0.8 P_treatment × age_ = 0.3
RBF (ml/kg/h)	883±113	826±38	936±142	492±13	P_treatment_ = 0.2 P_age_ = 0.017 P_treatment × age_ = 0.046
Filtration fraction (%)	15.2±0.8	20.9±0.9	8.4±1.2	19.0±2.1	P_treatment_ = 0.03 P_age_ = 0.002 P_treatment × age_ = 0.06
RVR (mmHg/ml/kg/h)	0.10±0.02	0.10±0.02	0.10±0.01	0.21±0.01	P_treatment_ = 0.008 P_age_<0.001 P_treatment × age_ = 0.001

UFR; urine flow rate, U_Na_V; Urinary sodium excretion, FE_Na_ %; fractional excretion of sodium, GFR; glomerular filtration rate, RBF; renal blood flow, RVR; renal vascular resistance. Values are mean ± SEM, from a two-way repeated ANOVA. n = 5 per treatment group per age.

### Measurements of Basal Mean Arterial Pressure and Heart Rate Over 72 Hours

Cardiovascular variables (MAP and HR) for the sleep and wake cycles were analysed and it was observed that the uni-x animals had elevated blood pressure compared to the sham animals at all times of the day, with no differences in heart rate observed between the treatment groups. The day/night difference in arterial pressure was similar in the uni-x and sham animals at all ages. These findings are similar to what has been previously reported in this model [28], therefore data has been averaged over 72 hours and are reported as cumulative averages in the current study. Continuous measurement of MAP in conscious animals revealed that MAP was similar between the treatment groups at 1 year of age but increased significantly in the uni-x group by 2 years of age. This increase in MAP was not exacerbated with ageing as no further increase in MAP was observed at 5 years of age (MAP: 1 year; sham: 78±2, uni-x: 79±1, 2 years; sham: 79±2, uni-x: 99±2, 5 years; sham: 80±1, uni-x: 101±1, P_treatment_<0.001, P_age_<0.001, P_treatment × age_<0.001). Heart rates were similar between the treatment groups at both ages and were not affected by ageing in either group (Heart rates (beats/min): 1 year; sham: 84±3, uni-x: 83±8, 2 year; sham: 79±3, uni-x: 85±5, 5 years; sham: 79±3, uni-x: 87±4, P_treatment_ = 0.2, P_age_ = 0.8, P_treatment × age_ = 0.4).

### Cardiovascular Variables Measured Over 24 Hours

**Figure 2:**
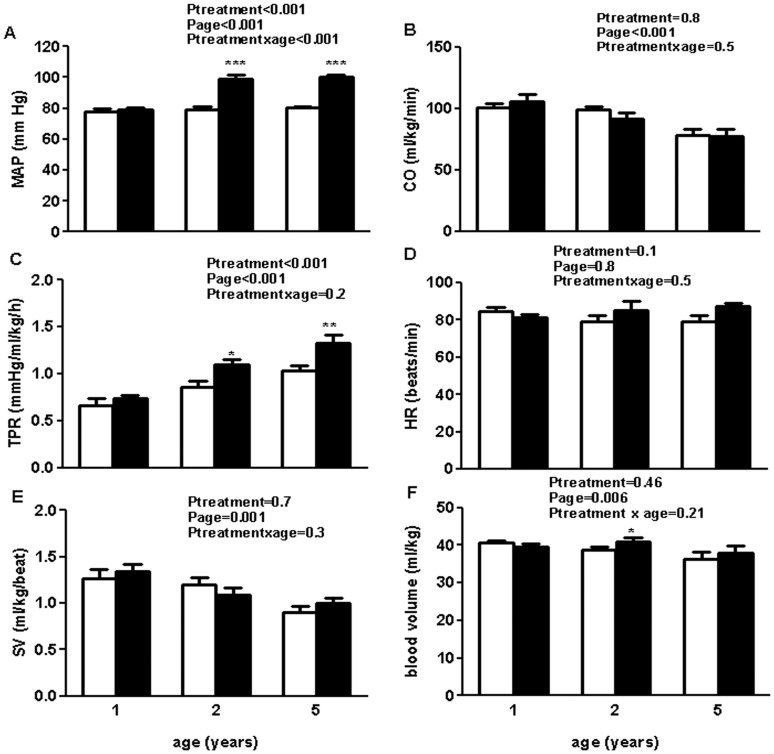
Basal cardiovascular 24 hour cardiovascular profile in sham-operated and uni-x female sheep at 1 year, 2 and 5 years of age. (A) Mean arterial pressure, MAP (B) cardiac output, CO (C) Total peripheral resistance, TPR, (D) Heart rate, (E) Stroke volume, SV and (F) blood volume. Values are from two-way repeated ANOVA and expressed as mean ± SEM. Sham (n = 5); clear bars, uni-x (n = 5); dark bars. *P<0.05, **P<0.01, ***P<0.001 as compared to sham group from post-hoc analysis.

Values for MAP (Fig. 2A) and HR (Fig. 2D) over 24 hours were not different to those obtained over 72 hours. CO was not different between the groups at any age, however, CO declined with ageing similarly in both treatment groups (P_treatment_ = 0.8, P_age_<0.001, P_treatment × age_ = 0.5, Fig. 2B). TPR was not different between the treatment groups at 1 year of age. With increasing age, TPR increased in both treatment groups (P_age_<0.001), with uni-x animals having a higher TPR compared to the sham animals at 2 and 5 years of age (P_treatment_<0.001). Post-hoc analysis showed that the increase in TPR was significantly greater with ageing in the uni-x animals at 5 years of age compared to the sham group (P<0.05 from Bonferroni post-hoc test, Fig. 2C). SV was not different between the groups at any age. However, SV declined similarly with ageing in both treatment groups (P_treatment_ = 0.7, P_age_ = 0.001, P_treatment × age_ = 0.3, Fig. 2E). A two-way repeated ANOVA revealed a significant effect of age on blood volume (P_age_ = 0.006), but revealed no effect of treatment (P_treatment_ = 0.46) or an interaction between age and treatment on blood volume (P_treatment × age_ = 0.21). A post-hoc analysis showed that at 2 years of age, uni-x animals had significantly higher blood volume than sham animals (P<0.05, Fig. 2F). Hematocrit levels were also similar between the treatment groups at 1 year and 2 years (P_treatment_ = 0.5), however, at 5 years of age, hematocrit levels had declined significantly but similarly in both treatment groups (P_age_<0.001, P_treatment × age_ = 0.96, Hct (%): 1 year; sham: 30.6±0.7, uni-x: 31.2±1.7, 2 years; sham: 30.4±0.6, uni-x: 31.2±2.1, 5 years; sham: 24.2±0.9, uni-x: 25.4±1.1). CVP was not different between the treatment groups at any age (CVP: 1 year; sham: 6.22±0.89, uni-x: 5.62±0.67, 2 years; sham: 6.36±0.29, uni-x: 5.80±0.20, 5 years; sham: 6.25±0.37, uni-x: 6.00±0.32, P_treatment_ = 0.4, P_age_ = 0.9, P_treatment × age_ = 0.9). PAM was similar between the treatment groups at 1 year and 2 years of age but became significantly elevated in the uni-x animals at 5 years of age compared to the sham animals. Post-hoc analysis revealed a significant increase in PAM with age in the uni-x animals only; P<0.05, (PAM: 1 year; sham: 13.7±0.5, uni-x: 14.1±0.8, 2 year; sham: 13.0±1.0, uni-x: 14.8±1.0, 5 years; sham: 14.5±0.9, uni-x: 17.4±0.6, P_treatment_ = 0.02, P_age_ = 0.03, P_treatment × age_ = 0.3).

### Cardiac Functional Reserve in Response to Dobutamine: Variables Corrected for Preload

Baseline values for MAP, CO, TPR, SV, HR, CVP and PAM measured over 2 hours were similar to those obtained over 24 hours at all the three ages studied (data not shown). Wedge pressure was similar in the uni-x and sham animals at 1 and 2 years of age but tended to be higher in the uni-x animals at 5 years of age compared to the sham group (P<0.05, from post-hoc analysis); (Wedge pressure (mmHg): 1 year; sham: 7.4±1.2, uni-x: 8.5±1.4, 2 year; sham: 7.2±1.4, uni-x: 8.3±0.3, 5 years; sham: 7.8±0.5, uni-x: 9.8±0.3, P_treatment_ = 0.007, P_age_ = 0.06, P_treatment × age_ = 0.5).

### Cardiac Functional Reserve in Response to Dobutamine at 1, 2 and 5 Years of Age

**Figure 3:**
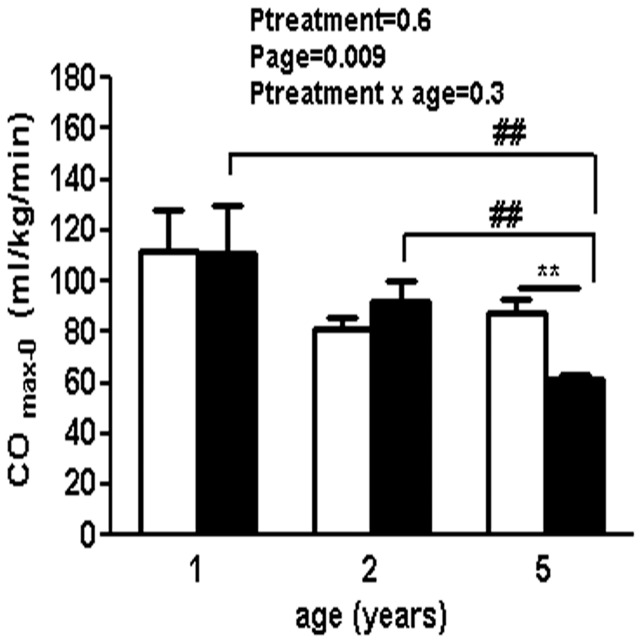
Cardiovascular response to dobutamine challenge in sham operated and uni-x female sheep at 1 year, 2 and 5 years of age following fetal unilateral nephrectomy at 100 d gestation. Cardiac functional reserve (CO _max−0_) is presented as maximal change in cardiac output (CO) compared to baseline (0). Values are from two-way repeated ANOVA and expressed as mean ± SEM. Sham (n = 5); clear bars, uni-x (n = 5); dark bars. **P<0.01 as compared to sham group at 2 years of age from post-hoc analysis. ##P<0.01 as compared to uni-x group values at 1 and 2 years of age from post-hoc analysis.

CO_max-0_ was similar between the treatment groups at 1 year and 2 years of age. However, at 5 years of age, uni-x animals had a significant reduction in CO_max−0_ compared to levels at younger ages and compared to the levels of the 5 year old sham animals (P<0.01 compared to 1 and 2 years of age and compared to sham at 5 years of age from post-hoc analysis, Fig. 3).

### Echocardiography at 2 and 5 Years of Age

Measurements for left ventricular dimension are shown in Table 2. LV mass (g) was not different between the treatment groups at 2 years of age. However, a significant increase in LV mass was observed in the uni-x animals with ageing (P_age_<0.001) compared to the sham group at 5 years of age, (P_treatment_<0.001, P_treatment × age_<0.001). There was no difference in LVEDd between the treatment groups at 2 years of age but uni-x animals had an increase in LVEDd at 5 years of age compared to the sham group (P_age_ = 0.002, P_treatment_<0.001, P_treatment × age_ = 0.001). PWTd was similar between the treatment groups at 2 years of age, but was greater in the uni-x animals compared to the sham group at 5 years of age due to an increase in PWTD with age in the uni-x group (P_treatment_<0.001, P_age_ = 0.06, P_treatment × age_<0.0001). IVSDd was not significantly different between the treatment groups at 2 years of age but a significant increase in IVSD was observed in the uni-x group at 5 years of age resulting in a greater IVSD compared to 5 year sham animals (P_treatment_ = 0.07, P_age_ = 0.0005, P_treatment × age_ = 0.006). % Fractional shortening was not significantly different between the treatment groups (P_treatment_ = 0.08), however, fractional shortening in the treatment groups was significantly dependent on age (P_treatment × age_ = 0.02). At 5 years of age, uni-x animals had a significant reduction in fractional shortening compared to age-matched sham group (P = 0.01 compared to 5 year sham group from after post-hoc analysis). There was no change in fractional shortening with age in the sham group, whilst uni-x animals tended to have a reduction in fractional shortening with age (P = 0.09, compared to 2 years, from post-hoc test). RWT was also similar between the treatment groups at both ages and did not change with age (P_treatment_ = 0.60, P_age_ = 0.9, P_treatment × age_ = 0.48, data not shown).

**Table 2:** Echocardiography results at 2 and 5 years of age in sham and uni-x animals.

	2 years		5 years		
Females	sham	uni-x	sham	uni-x	P-value
LVEDd (cm)	3.33±0.03	3.47±0.23	3.31±0.12	4.05±0.19	P_treatment_<0.0001 P_age_ = 0.002 P_treatment × age_ = 0.001
PWTd (cm)	0.78±0.01	0.82±0.04	0.72±0.09	1.01±0.11	P_treatment_<0.001 P_age_ = 0.06 P_treatment × age_<0.0001
IVSDd (cm)	0.93±0.03	0.97±0.05	1.01±0.11	1.20±0.09	P_treatment_ = 0.07 P_age_ = 0.0005 P_treatment × age_ = 0.006
LV mass (g)	112.1±2.6	118.1±10.8	109.7±10.92	173.1±25.0	P_treatment_<0.001 P_age_<0.001 P_treatment × age_<0.001
LV mass indexed (g/kg)	1.85±0.07	1.82±0.17	1.96±0.28	3.35±1.01	P_treatment_ = 0.001 P_age_<0.001 P_treatment × age_ = 0.001
Fractional shortening (%)	32.09±4.06	30.88±3.34	34.3±3.3	26.7±4.8	P_treatment_ = 0.08 P_age_<0.57 P_treatment × age_ = 0.02

LVEDd, left ventricular diameter in diastole; PWTd, posterior wall thickness in diastole; IVSDd, interventricular septum in diastole. LV, left ventricular mass; LV mass indexed, left ventricular mass corrected for body weight. Values are from two-way repeated ANOVA and expressed as mean ± SEM. n = 5 per treatment group per age.

### Cardiac Gene Expression at 5 Years of Age

Relative expression of collagen I, elastin and TGF-β1 were not statistically different between the treatment groups at 5 years of age (**collagen I**; sham: 1.07±0.18, uni-x: 1.56±0.18, P = 0.09, **elastin**; sham: 1.04±0.15, uni-x: 1.45±0.26, P = 0.2, **TGF-β1**; sham: 1.05±0.16, uni-x: 1.05±0.14, P = 0.9, P values from t-test).

### Collagen Content at 5 Years of Age

**Figure 4:**
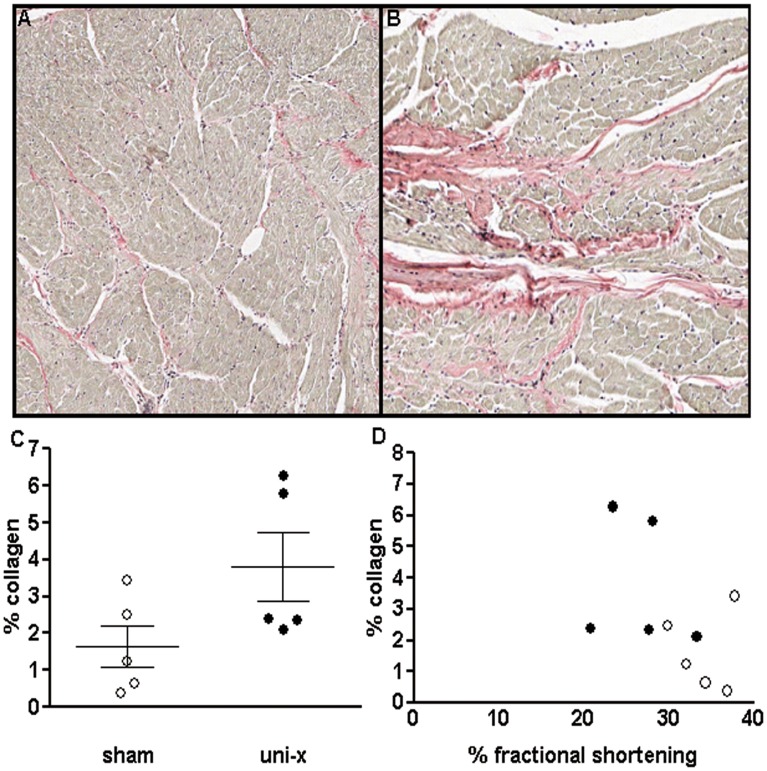
Photomicrographs of representative heart sections (5 µm paraffin sections) of female sheep at 5 years of age showing areas of collagen deposition (in pink) (A) sham (B) section from a uni-x heart showing significant collagen deposition (C) collagen content expressed as percentage of total area of cardiac tissue analysed. (D) Correlation between collagen content and percentage fractional shortening. Values are mean ± SEM. Sham (n = 5); clear circles, uni-x (n = 5); dark circles.

Percentage of interstitial collagen varied significantly within the treatment groups and was not significantly different between the sham and uni-x animals at 5 years of age (P = 0.08, student’s t-test, Fig. 4C). However, 2 uni-x animals exhibited areas of dense collagen deposition (Fig. 4B) that was not observed in any of the sham animals (Fig. 4A). There was no significant correlation between percentage collagen and percentage fractional shortening (Fig. 4D) and neither was there a significant correlation between LVmass and percentage collagen (data not shown).

## Discussion

The present study has shown that while intact uni-x female sheep exhibit a similar reduction in kidney function to our previous cohort of OVX uni-x female sheep [27], [28] they do not develop an elevation in blood pressure until 2 years of age which is in contrast to the previous observations of elevation in arterial pressure from 6 months of age in OVX uni-x female sheep [27], [28]. Interestingly, neither the decline in GFR nor elevation in arterial pressure was exacerbated with ageing. In addition, this study shows that uni-x female sheep had significant cardiac pathophysiology as indicated by reduced cardiac functional reserve at 5 years of age which was accompanied by left ventricular hypertrophy at this age.

The onset of hypertension was delayed in the intact uni-x female sheep in the present study, with elevations in blood pressure not observed until 2 years of age. The onset of hypertension in this study is associated with an elevation in blood volume in the uni-x animals at 2 years of age supporting Brenner’s hypothesis about the association between reduced nephron number, sodium retention and elevation in blood volume, thus blood pressure [35]. In this study while not statistically different, tubular sodium excretion was lower in the uni-x animals at 1 year of age and may have caused sodium retention over-time resulting in the elevation in blood volume and the increase in blood pressure observed in these animals from 2 years of age. These findings are similar to our observations in uni-x male sheep, in which urinary sodium excretion was significantly reduced contributing to an elevation in blood volume and an increase in arterial pressure, however in contrast to the present study, these phenotypes were observed in male sheep at the young age of 6 months [24]. In humans, the incidence of hypertension is greater in men compared to age-matched premenopausal women [1] however, these differences disappear post-menopause when rates of cardiovascular disease in women surpass that in men [3] and maybe associated with a decline in endogenous estrogen levels [36]. Estradiol levels show considerable variation both seasonally (as ewes have anoestrus for up to 6 months of the year) and also during the normal estrous cycle. Merino sheep enter an anestrus period in Spring early-Summer [37]. The pregnant ewes used in the current study were mated during Autumn-Winter period resulting in birth of offspring used in the present study to be over Spring-Summer. Taking into account that a 5 year old sheep is still within its reproductive years (prime reproduction age being 3–6 years), it is expected that the animals of this cohort would have undergone periods of estrus (Winter-Fall) and anestrus (Spring-Summer), similar to other sheep. The ages at which the studies were performed in the present study fell within the spring-summer period and whilst in the present study plasma estradiol levels were detected in some animals above the level of assay (∼3–4 pg/ml) at 1 year and 5 years of age, a study performed in Australian merino’s found that plasma estradiol levels are ∼7–8 pg/ml at the onset of estrus and peak at 10–15 pg/ml during the estrous cycle [38]. Therefore, plasma estradiol levels that were detected in some animals at their respective ages are below the aforementioned values indicating all animals were in anestrus at time of study.

The presence of estrogen may have delayed the onset of hypertension and similar levels across age may explain the lack of exacerbation in level of blood pressure in the uni-x females. It is noteworthy that OVX itself does not cause blood pressure to increase in sheep [39], but an increase in frequency of hypertension has been observed in OVX female rats in the Goldblatt two-kidney [40], one-clip model of hypertension and in the Ren2 rat [41]. We cannot discount there may be other differences besides the OVX between the current and the previous female sheep but birth weights, growth and the degree of renal insufficiency in the uni-x animals is similar in the two studies (approximately 30% decrease in GFR). Thus this study demonstrates that ovarian hormones may attenuate the development of hypertension in agreement with a previous study in rats^16^ but further highlights that a low nephron endowment confers a significant risk for the development of adult onset hypertension in both sexes as observed in numerous animal models [5], [6], [7], [11], [42], [43], [44] and in children with solitary kidneys [45].

In the current study, intact uni-x female sheep exhibited ∼30% reduction in GFR at 1 year of age, however, there was no further reduction in GFR with age in either the sham or the uni-x group. In our previous studies, female uni-x sheep that underwent OVX had a ∼30% reduction in GFR from 6 months of age [27] and a further ∼20% decrease in GFR was observed in both the sham and uni-x OVX sheep by 2 years of age [28]. At 2 years of age, OVX uni-x females also had a significant reduction in renal reserve [28]. The present findings also differ from our recent observations in male sheep, where an age-related decline in GFR was observed in both the sham and uni-x groups [46]. GFR has been shown to decline with ageing, commencing between 30–40 years with an accelerated decline observed after 50–60 years in humans [47] and this age-related decline in GFR has been documented to be blunted in women [48] and in female rats [49] compared to age-matched male counterparts. Furthermore, OVX in rats results in severe reduction in renal functional reserve and tubular fluid handling with ageing and these effects were prevented by estrogen administration [50]. Taken together, these observations suggest that estrogen may play a significant role in preventing the age-related decline in renal function in females as observed in the current study.

In some studies, preservation of GFR with age has been attributed to a preserved renal blood flow [48], [49]. While GFR was preserved in the uni-x animals with age, renal blood flow declined significantly between 1 and 5 years of age. The 30% drop in total GFR at 1 year of age is in conjunction with the estimated 30% reduction in nephron number in the uni-x animals in this model [51] and suggests that single nephron filtration rates are not different in the uni-x animals, suggesting absence of glomerular hyperfiltration, as compared to the sham sheep at 1 year of age. However, the reduction in filtration fraction in the uni-x animals indicates that single nephron blood flow is greater in the uni-x animals which may have occurred to maintain single nephron glomerular filtration rates by either a concurrent constriction of the afferent arteriole and dilation of the efferent arteriole and/or a decrease in the glomerular capillary ultrafiltration coefficient [52], which would reduce GFR for a given level of RBF. However, at 5 years of age filtration fraction between the uni-x and sham animals was similar due to a significant increase in filtration fraction in the uni-x animals with age. This is due to renal blood flow now being reduced to a similar extent to GFR in the uni-x animals. The increase in filtration fraction in the uni-x animals with age does indicate some degree of glomerular hyperfiltration, however total GFR still remained lower in the uni-x animals. We hypothesise that this suggests there has been further nephron loss in these animals with age and thus renal function has not been compensated for by the remnant nephrons. The reduction in renal blood flow in the uni-x female sheep with age maybe associated with the increase in renal vascular resistance and arterial pressure and is consistent with observations in uni-x male sheep in which renal blood flow was reduced at 6 months of age while renal vascular resistance and arterial pressure were elevated [25]. Certainly, renal vascular resistance has been documented to increase with ageing in normotensive individuals with the increase being exacerbated in hypertensive individuals [53]. The measurements in the present study are of total GFR and RBF and the normalisation of renal blood flow at 1 year of age in the uni-x sheep suggests that blood flow to the single kidney is approximately twice that going to each kidney in the sham animals and maybe associated with increased nitric oxide (NO) bioavailability as estrogens can directly induce NO production [54]. Unfortunately a caveat to this supposition is that renal blood flow was not determined in our previous cohort of OVX uni-x animals, thus we cannot be certain that the age-related decline in GFR observed by 2 years of age in the OVX uni-x animals [27], [28] was due to a reduction in renal blood flow. Conversely, the decline in RBF at 5 years of age in the present study maybe associated with a reduction in NO bioavailability [36] as estrogen has been shown to enhance NO bioavailability in normotensive but not hypertensive rats [55]. Another possibility maybe an increase in vasoconstrictors such as angiotensin II [56]. In the present study plasma renin activity was lower in the uni-x animals consistent with observations in OVX uni-x female sheep [27]. Studies have shown that in some models of low renin hypertension the intrarenal renin angiotensin system is inappropriately activated [57], [58], however, we have recently shown that at least in male uni-x sheep, renal renin and AngII levels were reduced [26]. Sex-differences in the expression and function of the RAS have been reported [14], therefore whether the decrease in renal blood flow in the female sheep at 5 years of age is associated with a reduction in NO availability or an increase in the intrarenal RAS remains to be investigated. Furthermore, decreases in RBF have also been associated with a decrease in renal size (which may reflect a decrease in nephron number) [47] and a decrease in nephron number with ageing has been documented previously [59]. Therefore, it is possible the uni-x female sheep have lost more nephrons with age, which is contributing to a decline in RBF: this also needs further investigation.

In the present study, echocardiographic measurements showed that while cardiac structure was normal at 2 years of age in the intact uni-x female sheep, a finding similar to that in OVX uni-x female sheep [28], at 5 years of age, intact uni-x female sheep had significant increases in left ventricular dimensions resulting in an increase in left ventricular mass and a decrease in fractional shortening. Furthermore, in response to a β-adrenergic challenge, cardiac functional capacity was preserved in the uni-x animals at 1 and 2 years of age, but was significantly reduced at 5 years of age compared to the sham group. It is likely that the enlarged ventricular mass and reduced fractional shortening maybe contributing to a reduction in cardiac contractility [60] and the reduction in cardiac functional reserve observed in the female sheep at this age. A reduction in cardiac functional reserve and increase in left ventricular size has also been demonstrated in 7 year old female sheep with hypertension due to prenatal glucocorticoid exposure [32]. These observations in the intact female sheep are particularly interesting as in uni-x male sheep we have shown increased left ventricular wall thickness at 6 months of age [25] and increased left ventricular mass at 2 years of age [24] together with a reduction in cardiac function in response to β_1_ adrenergic stimulation from as early as 6 months of age [24]. Preservation of myocardial mass has been reported to be better in women than men [61] and certain forms of familial hypertrophic cardiomyopathies are also more severe in men than women [62]. Witt et al [63] have shown that pressure-overload in mice causes more severe hypertrophy in males than females which is associated with a greater increase in hypertrophic genes and genes regulating extracellular matrix and a greater decrease in genes regulating mitochondrial function in males compared to females. While the exact mechanisms are unclear, estrogens have been shown to inhibit matrix metalloproteinases in human fibroblasts [64] and thus can alter composition of the extracellular matrix. The current study found no changes in the expression of pro-fibrotic factor TGF-β1, or the extracellular matrix gene, elastin but collagen I expression tended to be increased in the uni-x animals. Furthermore, while also not significant, collagen content as determined by picrosirius red staining also tended to be increased with some uni-x animals exhibiting regions of increased interstitial collagen. These observations suggest that there may be some degree of pathological cardiac fibrosis in the uni-x animals at 5 years of age, which is likely associated with the elevation in arterial pressure in these animals from 2 years of age. The alterations in cardiac structure may also reflect altered cardiac development following fetal uni-x. The timing at which uni-x is performed in our model coincides with the commencement of rapid maturation of cardiac myocytes in sheep [65]. While we found no information available on the effects of fetal uni-x on extracellular fluid status, bilateral nephrectomy has been shown to decrease extracellular fluid, plasma and blood volumes in the fetal sheep [66]. Furthermore, Dalshaug and colleagues have reported that 100d ovine fetal hearts have reduced ability to increase blood flow in response to a pressure overload challenge [67]. Whether, the developing heart also has a reduced ability to respond to altered body fluid homeostasis that may have been induced by fetal uni-x and whether this has any effects on the developing cardiomyocytes that manifest into adult cardiovascular pathophysiology needs to be further investigated in this model.

### Conclusion

In intact female sheep born with a nephron deficit, the reduction in renal function precedes the onset of hypertension. Whilst, the present study suggests that the presence of estrogen may play a role in the delayed development of hypertension, it appears that the existing renal insufficiency, overtime, outweighs this protection. Observations in our current and previous studies [24] highlight that a reduction in nephron number from birth confers a significant disadvantage to future cardiovascular and renal health in both male and female sexes and reiterates the need for rigorous monitoring of the health of children suspected of having a low nephron number such as those born of low birth weight. Given the significant differences observed in the onset and progression of both renal and cardiovascular dysfunction in male and female sheep in this model, future studies need to examine the contribution of molecular, anatomical and physiological factors to these phenotypes, in order to advance our understanding of the mechanisms that contribute to the sex-differences in cardiovascular and renal pathophysiology.
